# Antibiotic adjuvants: synergistic tool to combat multi-drug resistant pathogens

**DOI:** 10.3389/fcimb.2023.1293633

**Published:** 2023-12-20

**Authors:** Vikram Kumar, Nusrath Yasmeen, Aishwarya Pandey, Anis Ahmad Chaudhary, Abdullah S. Alawam, Hassan Ahmad Rudayni, Asimul Islam, Sudarshan S. Lakhawat, Pushpender K. Sharma, Mohammad Shahid

**Affiliations:** ^1^Amity Institute of Biotechnology, Amity University Rajasthan, Jaipur, Rajasthan, India; ^2^Amity Institute of Pharmacy, Amity University Rajasthan, Jaipur, Rajasthan, India; ^3^INRS, Eau Terre Environnement Research Centre, Québec, QC, Canada; ^4^Department of Biology, College of Science, Imam Mohammad Ibn Saud Islamic University (IMSIU), Riyadh, Saudi Arabia; ^5^Center for Interdisciplinary Research in Basic Sciences, Jamia Millia Islamia, New Delhi, India; ^6^Department of Basic Medical Sciences, College of Medicine, Prince Sattam bin Abdulaziz University, Al-Kharj, Saudi Arabia

**Keywords:** antibiotic resistant, multi-drug resistant, antibiotic adjuvants, pathogens, bacteria

## Abstract

The rise of multi-drug resistant (MDR) pathogens poses a significant challenge to the field of infectious disease treatment. To overcome this problem, novel strategies are being explored to enhance the effectiveness of antibiotics. Antibiotic adjuvants have emerged as a promising approach to combat MDR pathogens by acting synergistically with antibiotics. This review focuses on the role of antibiotic adjuvants as a synergistic tool in the fight against MDR pathogens. Adjuvants refer to compounds or agents that enhance the activity of antibiotics, either by potentiating their effects or by targeting the mechanisms of antibiotic resistance. The utilization of antibiotic adjuvants offers several advantages. Firstly, they can restore the effectiveness of existing antibiotics against resistant strains. Adjuvants can inhibit the mechanisms that confer resistance, making the pathogens susceptible to the action of antibiotics. Secondly, adjuvants can enhance the activity of antibiotics by improving their penetration into bacterial cells, increasing their stability, or inhibiting efflux pumps that expel antibiotics from bacterial cells. Various types of antibiotic adjuvants have been investigated, including efflux pump inhibitors, resistance-modifying agents, and compounds that disrupt bacterial biofilms. These adjuvants can act synergistically with antibiotics, resulting in increased antibacterial activity and overcoming resistance mechanisms. In conclusion, antibiotic adjuvants have the potential to revolutionize the treatment of MDR pathogens. By enhancing the efficacy of antibiotics, adjuvants offer a promising strategy to combat the growing threat of antibiotic resistance. Further research and development in this field are crucial to harness the full potential of antibiotic adjuvants and bring them closer to clinical application.

## Introduction

The evolution and spread of multidrug-resistant (MDR) bacteria is a matter of global health concern in the 21st century. A WHO report of 2017 identifies ESKAPE pathogens (Enterococcus faecium, Staphylococcus aureus, Klebsiella pneumoniae, Acinetobacter baumannii, Pseudomonas aeruginosa, and Enterobacter species) as critical-priority pathogenic bacteria causing fatal antibiotic resistance (Tacconelli et al., 2018). The emergence and spread of MDR pathogens pose a significant threat to public health worldwide. Multi-drug resistance is a complex and multifaceted problem. MDR occurs due to various factors, including the overuse and misuse of antibiotics such as unnecessary prescriptions, inappropriate dosages, incomplete or prolonged treatment courses, a lack of proper and quick diagnostic measures (Cantón et al., 2013), inadequate infection prevention and control practices in health care facilities, poor adherence to hygiene, a lack of sanitation (Chinemerem Nwobodo et al., 2022), and the genetic adaptability of microbial species. Furthermore, routine use of antibiotics in livestock production and in farming and agriculture facilitates the emergence of drug-resistant strains that can spread to humans via the food web or chain (Aslam et al., 2021).

Traditional antibiotics, once hailed as miraculous treatments, are becoming increasingly ineffective against these resilient microbial strains (Smith & Johnson, 2023). Mechanisms which collectively contribute to the reduced susceptibility of drug-resistant pathogens to conventional antibiotics are: 1) production of enzymes like β-lactamases, which break down the molecular structure of antibiotics to render them inactive, is a crucial strategy utilized by the pathogens (Egorov et al., 2018); 2) development of efflux pumps by drug-resistant bacteria to actively expel antibiotics and reduce their concentration in their cells; 3) modification of antibiotic target site; 4) enzymatic inactivation due to bacterial mutations causing production of altered ribosomes or enzymes which can diminish the binding affinity of antibiotics rendering them ineffective (Munita & Arias, 2016). The continuous selective pressure exerted by antibiotics has led to the evolution and dissemination of resistant strains, rendering many commonly used antibiotics ineffective (Davies & Davies, 2010). Consequently, infections caused by these drug-resistant pathogens are associated with higher mortality rates in vulnerable populations. Also lead to increased healthcare costs, prolonged hospital stays, and consuming more resources, resulting in the loss of productivity and necessitating high-acuity care. In recent years, the emergence of extensively drug-resistant (XDR) and pan-drug-resistant (PDR) bacteria has further exacerbated the crisis, leaving clinicians with limited or no treatment options (Struelens, 1998; Ozma et al., 2022). Above all, a lack of new antibiotic development strategies and dependence on already available antibiotics further exacerbate the problem.

This growing crisis necessitates the development of novel strategies to enhance the efficacy of existing antibiotics and combat the rising tide of drug resistance. In order to extend the life and efficacy of the current antibiotic arsenal, such novel strategies should focus on 1) increasing awareness of antibiotic stewardship among all health care communities, 2) enhancing research and development facilities to improve antibiotic production (Prestinaci et al., 2015); and 3) finding ways to prolong the life span and efficiency of currently available antibiotics. Other approaches under investigation are whole genome sequencing, quorum quenching (QQ), viral phage therapy, monoclonal antibodies, drug repurposing, novel small-molecule antibiotics with a focus on biologics and non-antibiotic adjuvants, and complementary and alternative therapies (Uddin et al., 2021). All of these aim to preserve the healthy microbiota while at the same time working toward preventing infections and resistance. However, among all of these strategies, using “antibiotic adjuvants in combination with antibiotics” has proven to be the most successful and effective (González-Bello, 2017).

Antibiotic adjuvants have emerged as a promising approach to counteract multi-drug resistance and restore the efficacy of existing antibiotics. Adjuvants are compounds or substances that are co-administered with antibiotics to enhance their antimicrobial activity. This can be achieved by either directly inhibiting bacterial resistance mechanisms or by potentiating the effects of antibiotics (Gill et al., 2015). The use of antibiotic adjuvants to resist AMR has potential advantages over the development of entirely new antibiotics. Adjuvants can effectively enhance the potency of existing antibiotics. This is made possible by lowering the minimum inhibitory concentration of the antibiotic required to kill the bacteria and allowing for the preservation of currently available treatment options (Melander and Melander, 2017; Laws et al., 2019). Repurposing the antibacterial compounds rendered obsolete (Schweizer, 2019), (Boyd et al., 2021) opens up the possibility of developing numerous analogues as antibiotic adjuvants. Moreover, the combination of adjuvants with antibiotics has the potential to reduce the rates of bacterial mutations, which has the potential to slow the development of resistance because of the well-conserved putative bacterial target (Allen & Brown, 2019). This approach represents a synergistic strategy where the combined effect of the adjuvant and the antibiotic is greater than the sum of their individual effects (Gill et al., 2015). AAs are resistant to antibiotics due to their unique mechanism (Annunziato, 2019), and most importantly, there is no selection pressure as the compounds are nonantibiotic in nature (Melander & Melander, 2017). The antibiotic adjuvant strategy also has certain limitations, like the identification of compounds or substances with essential physicochemical properties that can be used as adjuvants and co-administered with antibiotics, which is a daunting task. Additionally, the drug-drug interactions between antibiotics and adjuvants need to be assessed to avoid deleterious effects. Moreover, to facilitate an efficient co-dosing regimen with optimal spatial and temporal delivery options, adjuvants and antibiotics must have pharmacokinetic and pharmacodynamic properties that are compatible with one another. (Melander & Melander, 2017; Chawla et al., 2022). Concisely, antibiotic adjuvants are essential to enhance the susceptibility of bacteria to antibiotics and combat MDR. (Dhanda et al., 2023).

In the fight against MDR infections, the development and implementation of antibiotic adjuvants is a crucial weapon that offers hope for promising treatment alternatives against the impending danger. The main objective of this review is to provide a brief overview of the alarming issue of multi-drug resistance. It also discusses the mechanisms by which bacteria are rendering antibiotics obsolete, as well as the latest advancements in the development of antibiotic adjuvants. Furthermore, this research tries to notify the gap in the existing scenario and establish links between various strategies that are required to overcome AMR and make a substantial impact on public health and patient outcomes.

## Mechanisms of antibiotic resistance

**Figure 1:**
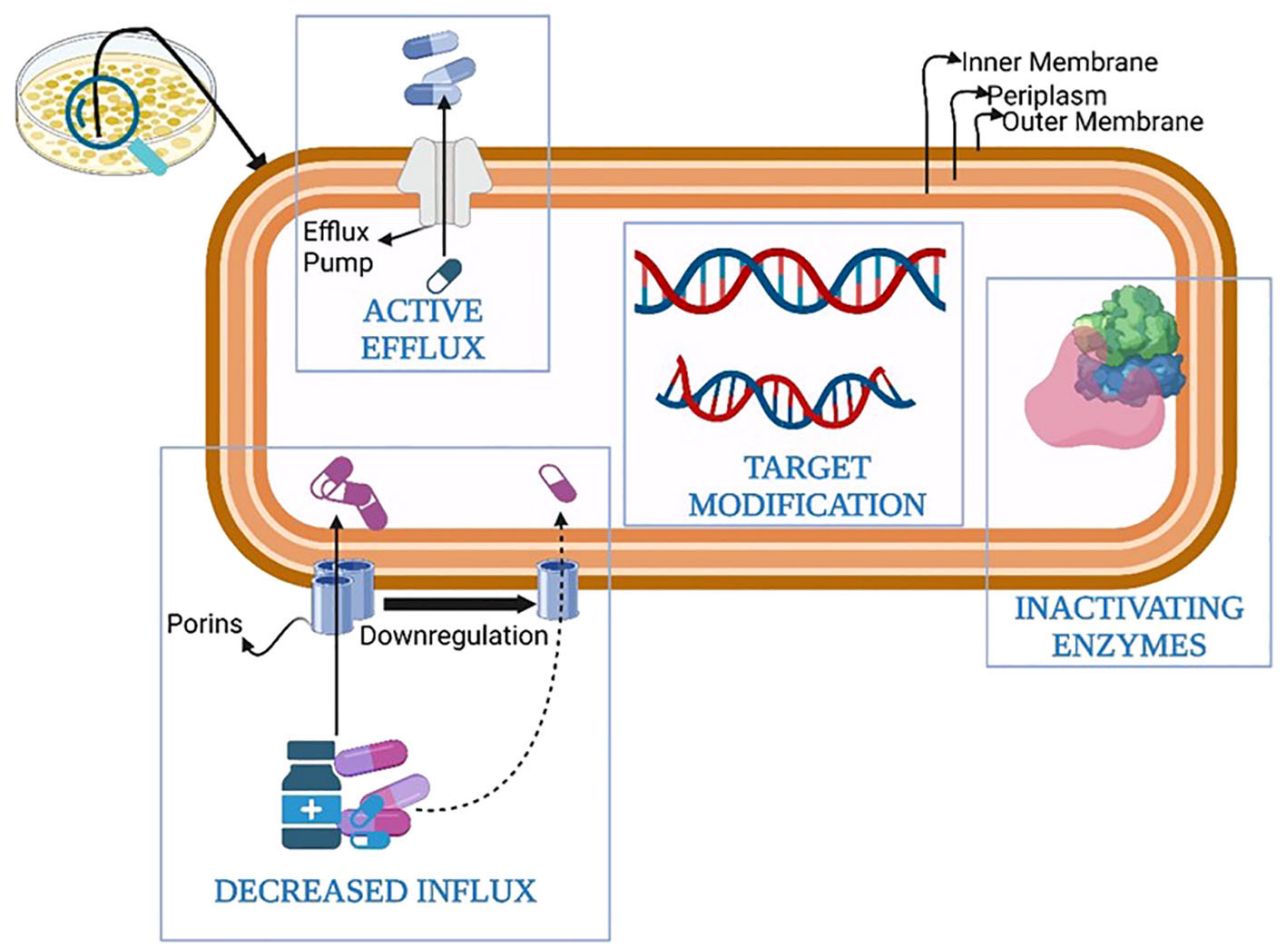
Antibiotic resistance mechanisms in pathogenic bacteria.

The development of defense mechanisms by bacteria against drugs that were once efficient at treating infections is known as antibiotic resistance (Blair et al., 2015). Bacterial defense armamentarium against antibiotics contributing to antibiotic resistance has evolved over time and includes the following methods: 1) genetic mutations; 2) modification of the drug target; 3) porin mutations causing a reduction in permeability; 4) increase in the number of efflux pumps; 5) enhance the secretion of inactivating enzymes and/or hydrolases; 6) changes in cell morphology; 7)Metabolic regulation or auxotrophy; 8) initiation of self-repair systems within the bacteria; 9) interaction between resistance protein and antibiotic target; 10) acquisition of resistance genes from other bacteria termed as community cooperative resistance; 11) biofilm formation and protection; 12) antibiotic avoidance (Hasan & Al-Harmoosh, 2020; Uddin et al., 2021; F. Zhang & Cheng, 2022). The most crucial among them are summarized in **Figure 1**.

### Genetic mutations

Bacteria can develop mutations in their DNA that result in changes in the structure of the antibiotic target, making it less susceptible to the drug. For example, some bacteria can develop mutations in their DNA that alter the structure of the ribosomes, which are the cellular structures responsible for protein synthesis. This can make it more difficult for antibiotics that target the ribosomes, such as macrolides and tetracyclines, to bind to their target and inhibit bacterial growth (Wang et al., 2023).

### Modification of the drug target

Bacteria can change the structure of their cell wall, membrane, or other cellular structures targeted by antibiotics, making them less susceptible to the drugs. For example, some bacteria can modify their cell wall structure, reducing the binding sites for antibiotics that target the cell wall, such as beta-lactams (Schaenzer & Wright, 2020).

### Porin mutations causing a reduction in permeability

The absorption of antibiotics by Gram-negative bacteria is significantly influenced by porin channels. Reduced permeability of multiple antibiotic classes, such as aminoglycosides and fluroquinolones, is caused by mutations that result in inactivation or downregulation of porin proteins, such as OprD (Reynolds et al., 2022).

### Increased expression of efflux pumps

Bacteria can have efflux pumps that can pump out antibiotics before they can reach their target inside the cell. These pumps can remove antibiotics from the bacterial cell and pump them back out into the environment, reducing the concentration of the drug inside the cell and making it less effective. Efflux pumps can be found in many different types of bacteria and can confer resistance to a broad range of antibiotics (Abdi et al., 2020).

### Enhance secretion of inactivating enzymes and/or hydrolases

Some bacteria produce enzymes that can break down or modify antibiotics, rendering them ineffective. For example, some bacteria can produce beta-lactamases, which are enzymes that can cleave the beta-lactam ring present in many antibiotics, including penicillins and cephalosporins, and make them inactive (Schaenzer & Wright, 2020).

### Changes in cell morphology

In the presence of antibiotics, bacteria can undergo several morphological alterations, which include changes in cell size, shape, surface-to-volume (S/V) ratio, or curvature. For example, P. aeruginosa changes from rod-shaped to spherical cells in the presence of β-lactam antibiotics. Bacteria also tend to reduce the surface-to-volume ratio in the presence of antibiotics, thereby decreasing the antibiotic influx due to reduced porin and efflux pump expression, ultimately reducing intracellular antibiotic concentration and thus antibiotic resistance. On the other hand, an increase in the S/V ratio leads to a dilution of antibiotics (Ojkic et al., 2022).

### Metabolic regulation or auxotrophy

Horizontal gene transfer or genetic mutations in bacteria can lead to significant physiological changes that might be accompanied by alterations in metabolism. Evidence suggests that the accumulation of alarmone (p)ppGpp triggers downstream signaling of rRNA and metabolic processes, determines stress survival, and modulates the bacterial response to antibiotics. For example, ppGpp alarmone mediates vancomycin tolerance in Enterococcus faecalis and also enhances the emergence and existence of persister cells of M. tuberculosis. On the other hand, the presence of auxotrophic strains of bacteria alleviates bacterial sensitivity to antibiotics and reduces the bactericidal actions of antibiotics, thereby enhancing antibiotic resistance (Zhang & Cheng, 2022).

### Initiation of self-repair systems within the bacteria

Bacteria can enhance antibiotic resistance by producing certain proteins and expressing genes encoding these proteins. These proteins might be responsible for DNA repair, lipid trafficking, or modulating the expression of porins and efflux pumps. The disruption in the repair of the damaged DNA is impacted by a lower antibiotic concentration, which ultimately leads to antibiotic resistance. Sharma et al. (2017) reported that transcription factors MarR and MarA in enteric bacteria transform the expression patterns of porin channels and efflux pumps, thereby playing a crucial role in antibiotic resistance. In a similar study, Erlandson et al. investigated the existence of a self-resistance protein and its influence on antibiotic resistance in echinomycin-producing bacteria. Ecm16 is a structural homolog of UvrA (a nucleotide excision repair protein) with ATPase activity. They reported that the expression of Ecm 16 in E. coli makes it an echinomycin-resistant strain. Hence, targeting these self-repairing proteins is a novel strategy to overcome antibiotic resistance (Erlandson et al., 2022).

### Interaction between resistance protein and antibiotic target

Target protection is crucial to enhance antibiotic resistance. It involves the interaction between resistance protein and an antibiotic target, which prevents the antibiotic from blocking its target and suppresses the effect of the antibiotic. Based on the underlying protection mechanism involved, target proception can be classified into three types: 1) type I, where the drug is removed from the target sterically; 2) type II, where conformational changes are induced within the target that cause the drug to dissociate allosterically from the target; 3) type III, where conformational changes are induced in the target, enabling antibiotic targets to remain functional when bound to antibiotics. For instance, direct displacement of the drug tetracycline from its target, i.e., the 70S binding site of ribosomes, by tetracycline ribosomal protection proteins (TRPPs), facilitates target protection and hence enhances antibiotic resistance (Wilson et al., 2020), (Zhang & Cheng, 2022).

### Acquisition of resistance genes

Bacteria can acquire resistance genes from other bacteria through the process of horizontal gene transfer, which can occur through conjugation, transformation, or transduction. Resistance genes can confer resistance to a specific antibiotic or a group of antibiotics and can be located on plasmids, transposons, or other mobile genetic elements that can be transferred between bacterial cells (Shehreen et al., 2019).

### Biofilm formation and protection

Bacteria can form biofilms, which are protective layers of extracellular matrix that can shield them from the action of antibiotics. Biofilms can make bacteria less susceptible to antibiotics by reducing the penetration of the drug into the bacterial cell and by providing a physical barrier that can prevent the drug from reaching its target (Gebreyohannes et al., 2019).

### Antibiotic avoidance

Some bacteria can avoid exposure to antibiotics by entering a dormant state, known as persistence or tolerance, which makes them less susceptible to the drugs. In this state, the bacteria can survive without dividing or growing, allowing them to evade the action of antibiotics that target rapidly dividing cells (Hembach et al., 2019).

These mechanisms of antibiotic resistance, as presented in **Table 1**, can occur individually or in combination. The development of novel antibiotics and the prudent use of already-existing ones are crucial in the fight against antibiotic resistance and prevent spread of resistant microorganism.

**Table 1:** Various types of antimicrobial resistance mechanism.

Type of Resistance	Antimicrobial Agents	Resistance Mechanism	References
Altered Target	Vancomycin	Alteration of cell wall stem peptide	(Wilcox et al., 2019)
	Tetracycline	Ribosomal Protection	(Manavathu et al., 1990)
	Trimethoprim	New drug insensitive	(Huovinen et al., 1995)
	Rifampin	Alteration of RNA Polymerase	(Goldstein, 2014)
	β- Lactam antibiotics	Alteration of binding proteins	(Yousif et al., 1985)
	Aminoglycosides	Alteration of ribosomal proteins	(Hummel & Böck, 1989)
	Erythromycin	Methylation of ribosomal RNA	(Hummel & Böck, 1989)
	Sulfonamides	New drug insensitive	(Sköld, 2001)
Limitation of Drug Uptake	Glycopeptides	Thickened cell wall, no outer cell wall	(Tenover et al., 2001)
	β- Lactam antibiotics	Diminished permeability	(Yousif et al., 1985)
	Quinolones	Altered outer member proteins	(Hooper & Jacoby, 2015)
	Chloramphenicol	Reduced permeability of outer membrane	(Roberts & Schwarz, 2017)
Inactivating Enzymes	β- Lactam antibiotics	Production of β- Lactamase	(Yousif et al., 1985)
	Aminoglycosides	Production of phosphotransferase, acetyltransferase and nucleotidyl transferase	(Hummel & Böck, 1989)
	Chloramphenicol	Production of acetyltransferase	(Roberts & Schwarz, 2017)
	Fluoroquinolones	DNA gyrase modification	(Sweeney et al., 2022)
	Tetracyclines	Antibiotic modification, oxidation	(Manavathu et al., 1990)
Active Efflux of the drugs	Erythromycin	New membrane transport system	(Hummel & Böck, 1989)
	Fluoroquinolones		(Sweeney et al., 2022)
	Tetracyclines		(Manavathu et al., 1990)
Modification of Porins	β- Lactam antibiotics	No outer cell wall, Decreased numbers of porins	(Yousif et al., 1985)
	Tetracyclines		(Manavathu et al., 1990)
	Cephalosporins	Changed selectivity of porins	(Pfeifer et al., 2010)
	Carbapenems		(Codjoe & Donkor, 2017)

## Antibiotic adjuvants (AA)

During empirical treatment, physicians generally prescribe two or more antibiotics in order to broaden the spectrum, covering all potential bacterial infections. Evidence suggests that combination therapy exerts a more synergistic effect, resists bacterial resistance, and reduces mortality rates in contrast to medication administration using monotherapy. (Dhanda et al., 2023). For instance, the broader-spectrum antibiotic Bactrim^®^, which is a combination of trimethoprim and sulfamethoxazole, is available, and the usage of many such combinations remains a clinical mainstay (Kalan & Wright, 2011). Combination therapy is not just restricted to the combination of two or more antibiotics but also involves the use of bioactive chemical entities commonly known as antibiotic adjuvants to augment the potency of a primary antibiotic. Moreover, treatment of drug-resistant bacterial infections using antibiotic adjuvant combinations is regarded as a better treatment choice than using numerous antibiotics. Concisely, it can be stated that antibiotic adjuvants (an antibiotic and a chemical entity) are used as part of combination therapy. They are the compounds with little or no antibiotic activity that are co-administered with antibiotics. They potentiate antibiotic effectiveness and minimize or block bacterial resistance by synergistically acting with them. (Liu et al., 2019). However, the major difference between combination therapy and using antibiotic adjuvant is that in the latter situation, antibiotics can be used alone as monotherapy, whereas adjuvant has no activity of its own (Worthington & Melander, 2013).

## Types of antibiotic adjuvants

Antibiotic adjuvants (AA) act synergistically with antibiotics to enhance bacterial susceptibility to antibiotics. They also modulate the host’s immune response to enhance the overall therapeutic outcome. Several classes of adjuvants are known, such as efflux pump inhibitors, β-lactamase inhibitors, quorum sensing inhibitors, and adjuvants targeting bacterial cell wall synthesis and membrane permeability. However, depending on the putative bacterial target and the action carried out, AA can be broadly classified as Class I and Class II antibiotic adjuvants (Wright, 2016). Class I AA acts by targeting active or passive resistance mechanisms in bacteria; Class II AA potentiates antibiotic activity in the host (De Oliveira et al., 2020). Different types of antibiotic adjuvants, along with examples, are presented in **Table 2**.

**Table 2:** Various antibiotic adjuvants along with their mechanism of action and functions.

class of Antibiotic adjuvant	Subclass of antibiotic adjuvant	Other names	Mechanism of action	Examples	Functions	References
**Class I**	**Class 1A** **inhibitors of active resistance**	β-lactamase inhibitors	Inhibitors of serine and metallo-β-lactamases.	Clavulanic acid Sulbactam Avibactam Relebactam etc.,	Suppress antibiotic resistance directly	(González-Bello et al., 2020)
	**Class 1B** **inhibitors of passive resistance**	Efflux pump inhibitors	Inhibit efflux pumps (MFS, SMR, MATE, RND etc)	MC-207,110 [phenylalanyl arginyl β-naphthylamide (PAβN)]; Carbonyl cyanide-m-chlorophenylhydrazone (CCCP)	Indirectly suppress intrinsic antibiotic resistance by enhancing antibiotic concentration	(Sharma et al., 2019)
		PMF inhibitors	Inhibit PMF by dissipating the electric potential (ΔΨ) and the proton gradient (ΔpH)	Loperamide Polymyxin B, thioridazine, Carbonyl cyanide-m-chlorophenylhydrazone (CCCP)	Indirectly suppress intrinsic antibiotic resistance	(Mohiuddin et al., 2022)
		Membrane permeabilizers	Enhance membrane permeability thereby increasing antibiotic uptake	guanidinylated polymyxins, colistin, cerium oxide nanoparticles	Inhibits antibiotic resistance by increasing antibiotic concentration within the cell	(Ramirez et al., 2022)
		Biofilm formation inhibitors	Inhibits biofilm formation	N-acetylcysteine (NAC), Tween 80, D-amino acid, polyamine norspermidine, Dispersin B(DspB), DNase I and α-amylase	Enhances sensitivity of bacterial cells to antibiotics, prevents formation of biofilm	(Ghosh et al., 2020); (Srinivasan et al., 2021)
		Quorum quenchers	Disrupt bacterial communication system	Furanone C30, patulin, penicillic acid and extracts from garlic, cinnamaldehyde, tobramycin (antibiotic) + baicalin hydrate (QSI); vancomycin+ hamamelitannin (QSI)	Inhibit bacterial resistance by disrupting bacterial survival techniques such as communication and biofilm formation	(Kalia, 2013)
**Class II**	**Targeting host defense mechanism**	immune enhancers	Modulate host immune system	Antimicrobial peptides (AMPs), human cathelicidin, LL-37; Active vitamin D; Phenylbutyrate; E-5564; TAK-242.under investigation	Enhance host immune system by modulating hypoxia-inducible factor 1(HIF),phagocytosis	(Ahmed et al., 2020); (De Oliveira et al., 2020)

Class I can be further differentiated into Class IA and Class IB based on their mechanisms of action (Ahmed et al., 2023). Class IA adjuvants are also known as “inhibitors of active resistance,” as they are capable of directly inhibiting antibiotic resistance. They work by inactivating or modifying enzymes, efflux pump systems, or modified drug targets. Class IB adjuvants are also known as “inhibitors of passive resistance,” capable of potentiating antibiotic activity. They act by evading intrinsic and passive resistance mechanisms such as signaling and regulatory pathways, altering outer membrane permeability, evading biofilm formation, or altering physiology (Wright, 2016; Sheard et al., 2019). Class II AA, known as “host modulating adjuvants,” work by targeting host cellular processes to enhance antibiotic efficacy, like triggering an immune response or an increase in phagocytosis. It is noteworthy that the AAs of Class IA are the only clinically available adjuvants, whereas those of Class IB and Class II are under investigation (Wright, 2016; Dhanda et al., 2023). AA can be generically categorized as beta(β)-lactamase inhibitors, efflux pump inhibitors (EPIs), membrane permeabilizers, and anti-virulence compounds based on their commercial availability. (González-Bello, 2017; Annunziato, 2019; Abd El-Aleam et al., 2021). Here are some common types of antibiotic adjuvants:

### β-Lactamase inhibitors

β-lactam antibiotics such as penicillins and cephalosporins are often combined with β-lactamase inhibitors. β-lactamase is an enzyme produced by some bacteria that can degrade β-lactam antibiotics, rendering them ineffective. Examples of β-lactamase inhibitors include clavulanic acid, sulbactam, and tazobactam (Bush & Bradford, 2016).

### Efflux pump inhibitors

Efflux pumps are mechanisms utilized by bacteria to pump out antibiotics from within their cells, reducing the intracellular concentration of the drug and leading to resistance. Efflux pump inhibitors (EPIs) can block these pumps, preventing antibiotic expulsion and increasing intracellular drug levels. Examples of EPIs include phenylalanine-arginine β-naphthylamide (PAβN) and carbonyl cyanide m-chlorophenylhydrazone (CCCP) (Sharma et al., 2019).

### Synergistic combinations

Certain adjuvants can enhance the activity of antibiotics when used in combination. For example, the combination of trimethoprim and sulfamethoxazole (TMP-SMX) works synergistically by inhibiting sequential steps in bacterial folate synthesis, leading to enhanced antimicrobial efficacy (Minato et al., 2018).

### Metal ion chelators

Some adjuvants function by chelating metal ions that are essential for bacterial growth and virulence. For instance, ethylenediaminetetraacetic acid (EDTA) can chelate divalent cations like calcium and magnesium, disrupting bacterial cell membranes and increasing the susceptibility of bacteria to antibiotics (Finnegan & Percival, 2015).

### Biofilm disruptors

Biofilms are communities of bacteria encased in a protective matrix, making them highly resistant to antibiotics. Adjuvants that can disrupt biofilms, such as enzymes (e.g., dispersin B) or specific agents (e.g., DNase), can enhance antibiotic penetration and efficacy against biofilm-associated infections (Roy et al., 2018).

### Immunomodulators

Some adjuvants work by modulating the host immune response, thereby enhancing the efficacy of antibiotics. Immunomodulators can stimulate the immune system, increase phagocytosis, or promote the release of cytokines and chemokines, which can aid in bacterial clearance. Examples include interferons, granulocyte colony-stimulating factor (G-CSF), and immunostimulatory oligonucleotides (Coffman et al., 2010).

### Nanoparticles

Nanoparticles can be used as adjuvants to improve antibiotic delivery, stability, and efficacy. They can enhance antibiotic solubility, prolong drug release, and increase drug accumulation at the infection site. Various types of nanoparticles, such as liposomes, polymeric nanoparticles, and metallic nanoparticles, have been explored as antibiotic adjuvants (Zong et al., 2022).

It’s important to note that the use of antibiotic adjuvants may vary depending on the specific antibiotic, the type of infection, and the resistance mechanisms involved. Additionally, the development and use of adjuvants are ongoing areas of research, and new types of adjuvants may emerge in the future.

## Mechanism of action of antibiotic adjuvants

**Figure 2:**
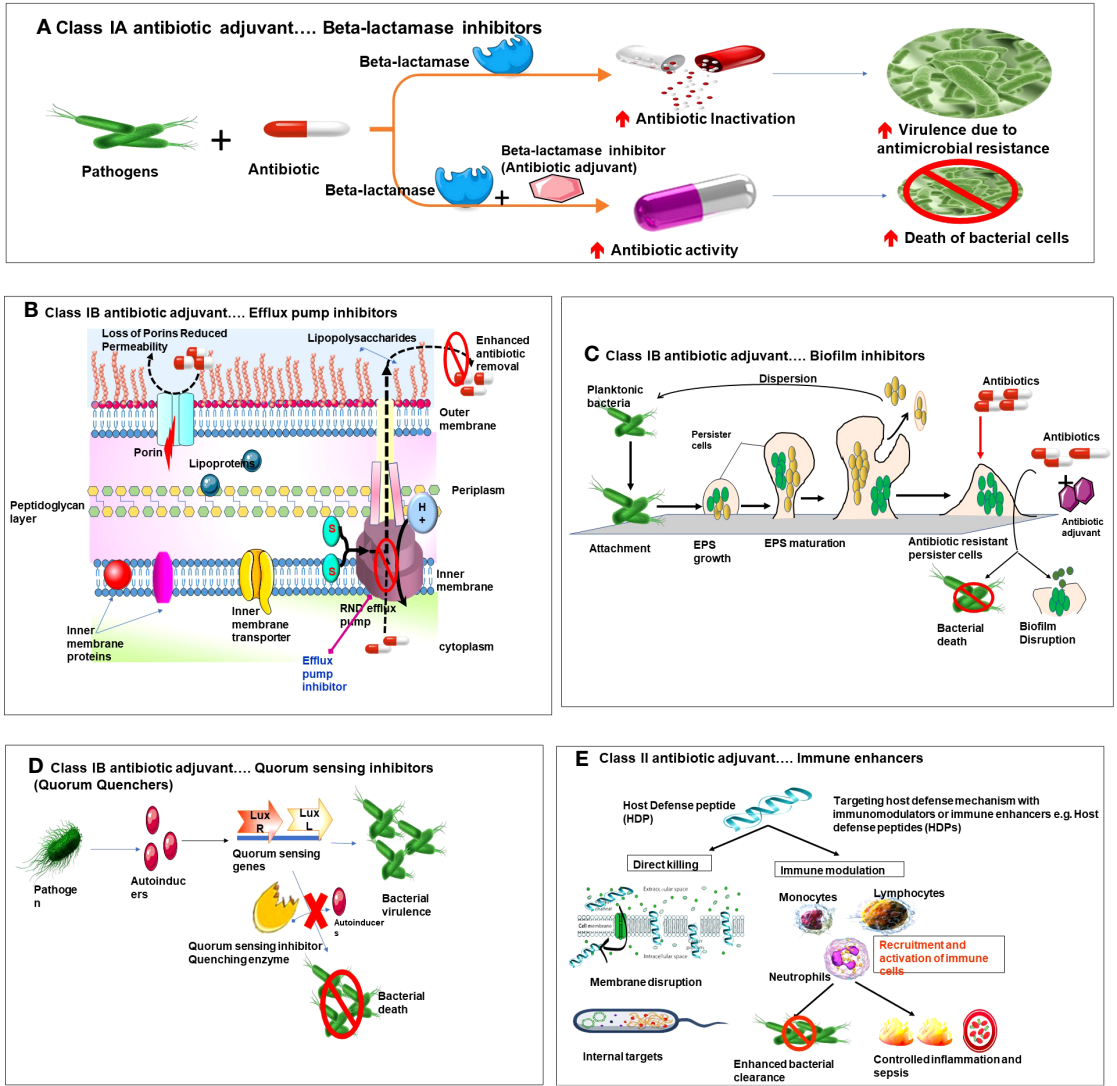
Diagrammatic representation of mechanism of action of various types of antibiotic adjuvants. **(A)** Class IA beta lactamse inhibitors. **(B)** Class IB Efflux pump inhibitors and membrane permeabilizers. **(C)** Class IB Biofilm inhibitors. **(D)** Class IB Quorum sensing inhibitors. **(E)** Class II Targeting host defense mechanism with immune enhancers.

AA enhance the antibiotic efficacy and halt/reverse the bacterial resistance via several mechanisms as depicted in **Figure 2**. Thus, they are also termed as “antibiotic potentiators” (Chawla et al., 2022) or “resistance breakers” (Laws et al., 2019) or “chemosensitizers”(Venter, 2019; González-Bello, 2017; Boyd et al., 2021). Different mechanism of action of antibiotic adjuvants as follows.

### Inhibitors of active resistance

Class 1A adjuvants act by targeting resistance caused by bacterial enzymes. Antibiotic activity is impaired by the resistance enzymes via multiple pathways, like modification of antibiotic targets, or antibiotic inactivation by hydrolysis or modification. Class 1A adjuvants are widely accepted adjuvants due to the clinical success exhibited by inhibition of enzyme -mediated resistance. The best example of this class of AA is Beta(β) lactamase inhibitors. Beta lactamase inhibitors are administered along with beta lactam antibiotics (penicillin, cephalosporins carbapenems and monobactams) (Idowu et al., 2019). These antibiotics possess a beta lactam ring which is essential for their antibiotic activity. However, β-lactamases are enzymes with potential to open this beta lactam ring and render the antibiotic ineffective(Tooke et al., 2019). In pursuit to overcome such resistance beta lactamase inhibitors were developed, they work by irreversibly binding to and inhibiting serine beta lactamases and metallo-β-lactamases, and hence preventing inactivation of the antibiotic. This combination (β-lactam antibiotic +β-lactamase inhibitor) is effective against wide spectrum of microorganisms (González-Bello et al., 2020).

The classical example of this combination widely used in clinics is Augmentin (Clavulanic acid + amoxicillin). Here, clavulanic acid irreversibly inhibits Ser-β-lactamases and retains the efficacy of antibiotic amoxicillin against the pathogens(Arer & Kar, 2023). Also, novel antibiotic adjuvants such as diazabicyclooctanones (DBOs), a non-β-lactam β-lactamase inhibitor used in combination with ceftazidime, imipenem and cilastatin show promising results (Yahav et al., 2020). Other examples of such combinations include tazobactam+ piperacillin; sulbactam+ ampicillin; avibactam +ceftazidime (Mosley et al., 2016); tazobactam+ ceftolozane; vaborobactam + meropenem (Lu et al., 2020; Principe et al., 2022). These inhibitors have broader spectra of activity and can inhibit a wider range of β-lactamases including extended -spectrum beta-lactmasese(ESBLs) and carbapenemases. However, inhibitors against carabapenem hydrolyzing oxacillinases (CHLDs) and New Delhi metallo-β lactamses(NDM-1) are under investigation.

### Inhibitors of passive resistance

Class 1B adjuvants have emerged as a promising strategy in the battle against antimicrobial resistance (AMR). They are also known as antibiotic potentiators or resistance breakers as they enhance the efficacy of currently available antibiotics, thereby potentiating their action against resistant bacteria.

### Efflux pump inhibitors (EPIs)

One of the main mechanisms by which class 1B adjuvants combat AMR is by inhibiting bacterial efflux pumps. Efflux pumps are cellular transport proteins which are actively involved in the cellular extrusion of substances, including antibiotics out of bacterial cell, leading to decreased concentration of antibiotic in the cell and thus reduced efficacy, also generating antibiotic resistance phenotype of bacteria that expresses efflux pump (Abdel-Karim et al., 2022). The types of efflux pumps that exist in these pathogens are i) ATP binding cassette (ABC), ii) small multidrug resistance(SMR) family, iii) major facilitator superfamily (MFS) iv) multidrug and toxin extrusion (MATE) family and v) resistance nodulation and cell division (RND) family and vi) proteobacterial antimicrobial compound efflux (PACE) superfamily (Huang et al., 2022). The RND superfamily is prominent efflux pump family in Gram -ve bacteria, whereas MFS, ABC, MATE and SMR are seen in both Gram +ve and Gram -ve bacteria (Blanco et al., 2016; Auda et al., 2020). Class 1b adjuvants such as EPIs can inhibit these efflux pumps, preventing antibiotic expulsion and allowing higher concentrations of antibiotics to accumulate within the bacterial cells, thereby increasing their potency (Abdel-Karim et al., 2022). Lomovskaya et al., were the first to develop a peptidomimetic EPI, named MC-207,110 [phenylalanyl arginyl β-naphthylamide (PAβN)]. They demonstrated that this EPI when used in combination with either levofloxacin or erythromycin enhances their antibacterial activity against MexAB-OprM-overexpressing strains of P.aeruginosa (Lomovskaya et al., 2001). Successively, several other EPIs were developed but unfortunately couldn’t reach the clinical realm due to toxicity issues in mammalian cells.

### Membrane permeabilizers

Class 1B adjuvants known as membrane permeabilizers or membrane saboteurs can enhance the permeability of bacterial cell membranes, facilitating the entry of antibiotics into bacterial cells, enhancing their intracellular concentration essential to kill the bacteria and to overcome resistance (Si et al., 2022). Hydrophobic antibiotics readily cross the lipid bilayer whereas hydrophilic antibiotics can enter bacteria only through channel forming proteins called porins (Gill et al., 2015). Many factors such as membrane lipids, membrane composition, porins and presence of efflux pumps influence membrane permeability and drug uptake affecting susceptibility of bacteria to antibiotics. Outer membrane(OM) of Gram -ve bacteria has an additional outer layer of polyanionic lipopolysaccharides (LPS) that makes the membrane more impermeable than Gram +ve bacteria (Farrag et al., 2019). Electrostatic interactions between divalent cations (Ca2+ and Mg2+) and anionic phosphate group in lipid A are essential for stabilization of OM LPS structure (Domalaon et al., 2018). Over expression of porins and extra outer membrane confer resistance to bacteria (Delcour, 2010). Antimicrobial peptides (Colisitn), detergents, nanoparticles (Polymyxin B nonapeptide (PMBN) can be used as membrane saboteurs to enhance membrane permeability and increase antibiotic concentration essential to kill the bacterial cell (Si et al., 2023). Cerium oxide nanoparticles were demonstrated to be potential antibiotic adjuvants specifically membrane permeabilizers that enhance the antibiotic activity against AMR (Bellio et al., 2018). Natural compounds such as thymol and gallic acid, glycosylated cationic block poly beta peptide (PAS8-b-PDM12) (Si et al., 2020), chitosan derivatives 2,6-diaminochitosan (2,6-DAC) (Si et al., 2021), glycine containing amino acid conjugated polymer (ACP) (Barman et al., 2019), polyurethanes and polycarbonates were found to be potent membrane permeabilizers.

### Proton motive force (PMF) inhibitors

Furthermore, for agents with cytosolic targets inner membrane acts as a potential barrier, restricts the diffusion of hydrophilic molecules. Transporter proteins are essential for movement of charged particles and hydrophilic molecules into the cell. Efflux pumps utilize proton motive force (PMF) to remove antibiotics, decrease their concertation and hence protect the bacterial cell (Wang et al., 2021). PMF is an electro chemical proton gradient governed by the electric potential (ΔΨ) and the proton gradient (ΔpH). A balance is maintained between these two components to have stable PMF levels (Farha et al., 2013). PMF is the driving force required for ATP synthesis in bacterial cell, it also powers bacterial cell motility and cell division (Lee et al., 2023). PMF is essential for entry of weakly charged and amphiphilic particles into cytosol. Tetracyclines and loperamide penetrate bacterial cell in a ΔpH-dependent manner, however, loperamide dissipates the electric potential (ΔΨ) of PMF. As a counter regulatory mechanism to maintain PMF maintain and ATP synthesis levels, bacteria increase the transmembrane proton gradient (ΔpH) in the cell which in turn enhances the uptake of tetracycline. In contrast, aminoglycoside uptake is ΔΨ dependent, explaining the antagonistic effect between loperamide and aminoglycosides (Ejim et al., 2011). Polymyxin inhibits ΔΨ and alters membrane permeability by disrupting LPS structure. PMF inhibitors e.g., thioridazine, nordihydroguaiaretic acid, trifluoperazine, gossypol, amitriptyline and carbonyl cyanide m-chlorophenyl hydrazone (CCCP),antibiotic hybrids were found to eliminate MRSA cells by disrupting PMF by exhausting either ΔΨ or ΔpH (Mohiuddin et al., 2022).

### Biofilm inhibitors

Bacterial growth adaptations against stress, desiccation, the oxidizing effects of chemicals, and the host’s immune system led to the development of bacterial biofilms. Concisely, these biofilms are essential for bacterial survival in harsh environments. In addition to contributing to antibiotic resistance and enhancing bacterial pathogenicity, they are the source of chronic or persistent infections. Bacteria tend to live in communities attached to the nearby surfaces enclosed within a matrix. This extracellular matrix (ECM) will be formed by bacterially secreted extrapolymeric substances (EPS), which are a complex mixture of exopolysaccharides, DNA, proteins, and lipids. These stationary microbial communities that thrive on host tissues and implanted medical devices are termed ‘Biofilms’. The biofilm matrix allows bacterial communities to co-exist in close proximity. These biofilm communities are heterogeneous and possess interstitial voids, a unique architectural feature. The main function of the interstitial voids is to act as a circulatory system and facilitate the diffusion of nutrients, gases, and antimicrobial agents, as well as the removal of metabolic wastes. The existence of cells in close proximity to biofilms enables the exchange of plasmids, quorum sensing molecules called quoromones, and horizontal transfer of antibiotic resistance genes (Ghosh et al., 2020; Davies and Davies, 2010).

The transition from the free-floating planktonic phase to the formation of biofilm is an intricate and tightly controlled multi-step cascade that occurs via the following steps: The first step consists of the attachment of planktic bacterial cells to the surface using appendages, sex pili, or fimbriae, under stress conditions. The second step involves the aggregation and colonization of single-seeded cells. Subsequently, growth and cell division of seeded bacterial cells occur, during which they secrete EPS that offers structural rigidity and release chemicals called quoromones (quorum sensing molecules), responsible for bacterial cell communication and controlling the gene expression of the biofilm. Cell division and aggregation/colonization occur until there is a significant increase in the cell density in the biofilms. This is called the maturation phase. This mature biofilm structure is responsible for antibiotic resistance. In this stage, the EPS restricts the movement of substances and increases toxic buildup, due to which the bacteria get activated. Subsequently, they secrete EPS-digesting hydrolases, which help dissolve EPS around them and release the planktic bacterial cells, which can seed into new colonies (Luo et al., 2021). Biofilm formation is crucial for the development of antibiotic resistance, recalcitrance, and recurrent chronic infections that are difficult to treat. For instance, biofilm-forming phenotypes of P. aeruginosa were found in the clinical isolates of cystic fibrosis patients, in patients with chronic wounds, and also in those suffering from ventilator-associated pneumonia (VAP), making it difficult to treat such patients (Mulcahy et al., 2014).

The mechanisms by which bacterial biofilms enhance antimicrobial resistance are: 1) resistance due to the complex nature of the biofilm surface; 2) resistance due to challenging microenvironments within the biofilm; and 3) resistance due to the polymorphic and heterogeneous nature of the biofilms (Luo et al., 2021). The EPS is a sticky layer that acts as a natural defense, shielding the bacteria against stressful conditions and chemicals. Its composition is also varied. EPS delays diffusion, decreases the rate of drug penetration, and blocks the entry of certain chemicals, thereby enhancing resistance to antibiotics. The microenvironment inside the biofilm is intriguing and prone to change. At different strata, it has different chemistry; deep inside, metabolic wastes and nutrients are accumulated. It becomes more anaerobic in nature, which is not favored by antibiotics. Some of the bacteria turn into spore-like dormant forms called ‘Persister cells (Abebe, 2020). These dormant forms do not divide in unfavorable conditions and are central to the recalcitrance of chronic cells, enhancing antibiotic resistance; hence, the eradication of persisting cells is also important (Allen & Brown, 2019). Antibiofilm agents, either alone or in conjunction with antibiotics, are being used to destroy bacterial biofilms. Many agents such as N-acetylcysteine (NAC), Tween 80, D-amino acid, polyamine norspermidine (Böttcher et al., 2013), Dispersin B (DspB), DNase I, and α-amylase are used as antibiofilm agents (Hawas et al., 2022). These agents can be classified under Class IB AA as they work toward enhancing antibiotic sensitivity by disrupting various phases in biofilm formation and maturation, such as i) inhibition of adherence of bacteria to surfaces; ii) attenuation of QS signaling cascade; iii) disruption of second messengers involved in various signaling cascades; iv) inhibition of biofilm maturation (Wu et al., 2015; Ghosh et al., 2020). v) modulation of cyclic di-guanosine monophosphate (c-di-GMP) or passive (enzymatic or physical biofilm disruption) essential for dispersal of mature biofilms (Wille & Coenye, 2020). Yu et al. (2015) showed that exogenously administered Ps1G, a glycosyl hydrolase produced by P. aeruginosa, effectively inhibits biofilm formation and enhances disruption of existing biofilms. Zhang et al. (2013) reported that streptomycin coupled with chitosan inhibited biofilm formation and led to the disruption of established biofilms.

### Quorum quenchers/quorum sensing inhibitors

Alternatively, bacterial co-existence in the host depends mainly on its chemical communication using specialized molecules called ‘auto-inducers (AIs),’ and this phenomenon is called “quorum sensing (QS)” (LaSarre & Federle, 2013). QS helps the bacteria suppress the host immune system, migrate to a suitable environment to flourish, and form biofilms; all these acts render antibiotics less efficient (Soukarieh et al., 2023). The molecules involved in QS are different in Gram-negative (-ve) and Gram-positive (+ve) bacteria. Based on their molecular structure, QS signaling molecules are divided into three groups: 1) QS molecules in Gram+ve bacteria are oligopeptides or auto-inducing peptides (AIPs). For example, in Staphylococcus aureus, AIPs are QS molecules used for chemical communication, where the autoinducer phosphorylates the receptor bound to the cell membrane. 2) Furthermore, QS signaling molecules in Gram-ve bacteria are derivatives of fatty acids such as N-acyl-homoserine lactones (AHLs) and cis-11-methyl-2-dodecenoic acid (DSF). For example, in Pseudomonas aeruginosa, AHLs synthesized by LuxI-type synthases or other molecules synthesized from S-adenosylmethionine (SAM) are used, where the autoinducers bind to cellular receptors after freely diffusing into the cell (Lazar et al., 2021). 3) Other classes of QS signaling molecules include autoinducer-2 (AI-2), α-pyrone, integrated quorum-sensing signal (IQS), 3-hydroxy-methyl palmitate (3-OH-PAME), Pseudomonas quinolone signal (PQS), dialkyl resorcinols (DARs), and p-coumaroyl-HSL (aryl-HSL) (Zhu et al., 2023). QS disruption is crucial to overcome antibiotic resistance. It could be possible by tampering with either the QS signaling molecules, such as AHLs, or interfering with the QS signaling pathways (modifying the signal generator or receptor). The enzymes used to disrupt QS signals are called quorum quenching enzymes (QQE). QS pathways can be modulated using chemicals known as quorum quenchers (QQ) or quorum sensing inhibitors (QSIs), which further decrease QS-controlled gene expression (Fong et al., 2018). Soukareih et al. reported that QSI (R)-2-(4-(3-(6-chloro-4-oxoquinazolin-3(4H)-yl) -2-hydroxypropoxy)phenyl)acetonitrile, conjugated with the comonomer 2-dimethylaminoethyl acrylate (DMAEA), could efficiently disrupt P. aeruginosa biofilms when used in combination with ciprofloxacin (Soukarieh et al., 2023).

### Targeting host defense mechanisms

AMR can be subdued by using unconventional treatment strategies where non-vital bacterial processes are targeted, such as cell attachment, host-pathogen interactions, or enhancing host defensive mechanisms. These mechanisms offer advantages such as lesser selection pressure and stable host protein targets due to the slow evolution of microbial resistance. Class II AA are a new class of drugs that are currently under investigation targeting the host’s defensive mechanisms, making them suitable candidates to overcome the existing antibiotic crisis. They can be termed “immune enhancers.” The host defense mechanisms being targeted include: i) regulating pattern recognition receptor (PRR) signaling pathways. e.g., 4C-Staph/T7-alum vaccine containing a TLR7-agonist (SMIP.7-10) and T7-alum adjuvant was found efficient to treat peritonitis in mice caused by staphylococcal strains (Bagnoli et al., 2015; Mancini et al., 2016); ii) enhancing the autophagy activity through the use of P38 and cathepsin B inhibitors. (C) Stabilizing hypoxia-inducible factor 1α (HIF α). HIF-1α is a key regulator of the immune response. Augmentation of HIF levels might enhance antimicrobial activities and boost infection clearance in the majority of infections. Contrastingly, some microorganisms, such as Toxoplasma gondii and Leishmania, thrive well when HIF levels are enhanced. Zinkernagel et al. (2008) reported that mimosine augments HIF-1α, which enhances the bactericidal activity of phagocytes. Similarly, it was found that HIF-1α stabilizing agent AKB-4924 reduced uropathogenic Escherichia coli (UPEC) bladder infection in mice by 10-fold; it was found to be efficient in killing Acinitobacter baumanii and Pseudomonas aeruginosa and limiting S. aureus in a skin abscess model in mice (Okumura et al., 2012). (D) regulating ROS and RNS production. ROS-inducing strategies like antimicrobial photodynamic therapy (aPDT) and cold atmospheric plasma (CAP) enhance microbial eradication used to treat wounds and in food contamination cases (Li et al., 2021). Alternatively, antibiotics, when combined with antimicrobial peptides, show synergistic effects and enhance antimicrobial activity. Haisma et al. (2014) demonstrated that peptides of human cathelicidin LL-37 and its derivatives, such as P60.4Ac and peptide 10 (P10), were efficient antibiofilm agents and could efficiently eradicate multidrug-resistant S. aureus (MRSA) and mupirocin MRSA from burn wounds without altering the viability of the host keratinocytes. Another example is that of BAY 11-7082 (BAY, an inhibitor of IkBα kinase), which inhibits nuclear factor-kappa B (NF-kB) activation, which could in turn enhance macrophage apoptosis and autophagy, which are essential for pathogenic clearance and killing intracellular Mycobacterium tuberculosis (MTB) (Bai et al., 2013). However, care must be taken while using immune enhancers, as overstimulation of the immune system can lead to deleterious effects on the host.

Finally, it is plausible to conclude that antibiotic adjuvants are crucial, with significant clinical ramifications in the fight against antimicrobial resistance. They can be a valuable addition to the arsenal of existing antibiotics due to their ability to enhance antibiotic efficacy, prevent biofilm formation, provide alternative strategies to overcome resistance, and prevent its further development. These valuable tools can be utilized in clinical practice for managing infections caused by multidrug-resistant pathogens; they can significantly improve prognosis and preserve the effectiveness of antibiotics in the long term.

## Conclusion and future prospective

Antibiotic adjuvants represent a promising and synergistic tool in the battle against MDR pathogens. The current study on antibiotic adjuvants opens up several avenues for future research and further directions. Here are some potential areas to explore: Adjuvant Development: With the rise of antibiotic resistance posing a significant threat to public health, the development of new strategies to enhance the efficacy of existing antibiotics is crucial. Adjuvants offer a unique approach by targeting the underlying mechanisms that contribute to antibiotic resistance and working in synergy with antibiotics to overcome these challenges. Continued research and development of adjuvants is crucial. Investigating new adjuvant compounds or modifying existing ones to improve their efficacy, safety, and specificity can lead to the discovery of more potent adjuvants. Understanding the mechanisms of action and identifying new targets for adjuvants can further enhance their ability to combat multidrug-resistant pathogens. Mechanism Elucidation of adjuvants: one of the key advantages of antibiotic adjuvants is their ability to reverse or suppress antibiotic resistance mechanisms in bacteria. Adjuvants can inhibit efflux pumps, enzymes, or biofilm formation, which are common strategies employed by bacteria to resist the effects of antibiotics. By blocking these resistance mechanisms, adjuvants can restore the susceptibility of bacteria to antibiotics, allowing the drugs to once again effectively kill the pathogens. Another important aspect of antibiotic adjuvants is their potential to target bacterial virulence factors. Unlike antibiotics that primarily target bacterial growth and survival, adjuvants can interfere with the mechanisms that enable bacteria to cause harm to the host. By inhibiting virulence factors such as toxin production or adhesion to host tissues, adjuvants can attenuate the pathogenicity of bacteria, allowing the host’s immune system and antibiotics to more effectively clear the infection. Gaining a deeper understanding of the mechanisms by which adjuvants overcome antibiotic resistance is essential. This knowledge offers a significant advantage over developing entirely new antibiotics or adjuvants with tailored mechanisms of action for different resistance mechanisms or bacterial species. Furthermore, antibiotic adjuvants have demonstrated the ability to enhance the activity of antibiotics through synergistic interactions. Adjuvants can potentiate the effects of antibiotics by increasing their uptake into bacterial cells, improving their binding to targets, or interfering with bacterial defense systems. This synergistic effect not only improves the overall efficacy of antibiotics but also helps to overcome the development of resistance. Combination Strategies: By using adjuvants in combination with antibiotics, lower doses of antibiotics can be used, reducing the likelihood of resistance emergence. Investigating the potential of combining adjuvants with other therapeutic approaches, such as phage therapy, immunotherapy, or host-directed therapies, is a promising direction. Understanding the synergistic effects and potential interactions between adjuvants and alternative therapies can provide valuable strategies for combating multidrug-resistant pathogens. *In vivo* studies: Identifying adjuvants with high specificity and low toxicity is essential to ensuring their safety and efficacy. Additionally, the optimization of adjuvant-antibiotic combinations requires extensive research, both preclinical and clinical trials in animal and human models, to determine the most effective strategies for different pathogens and resistance mechanisms. Clinical implementation: Translating the use of adjuvants into clinical settings is a crucial step. Real-world studies can provide data related to cost effectiveness, feasibility, toxicity, etc., which can provide protocols necessary for their usage in clinical settings. However, it is worth noting that the development and implementation of antibiotic adjuvants face certain challenges like lack of proper research, insufficient funds, rapidly growing resistant strains, etc. Despite these challenges, antibiotic adjuvants hold great potential for combating MDR pathogens. They offer a multifaceted approach by targeting resistance mechanisms, enhancing antibiotic activity, and attenuating bacterial virulence. The integration of adjuvants into existing antibiotic treatment regimens could revitalize the efficacy of currently available antibiotics, prolong their lifespan, and provide an essential tool in the fight against antibiotic resistance. Moving forward, continued research and development in the field of antibiotic adjuvants are crucial. Collaborations between scientists, clinicians, and pharmaceutical companies are necessary to identify and optimize adjuvants that can be successfully translated into clinical practice. Additionally, regulatory agencies play a vital role in ensuring the safety and efficacy of adjuvants and providing guidelines for their appropriate use.

In conclusion, antibiotic adjuvants offer a promising solution to combat MDR pathogens. By addressing the challenges posed by antibiotic resistance through various mechanisms, adjuvants have the potential to revolutionize the treatment of infectious diseases. Continued efforts to develop and implement antibiotic adjuvants are essential in our fight against the growing threat of antibiotic resistance and to safeguard public health.

## Author contributions

VK: Conceptualization, Supervision, Writing – original draft, Writing – review & editing. NY: Conceptualization, Data curation, Investigation, Methodology, Software, Validation, Writing – original draft, Writing – review & editing. AP: Writing – review & editing. AC: Funding acquisition, Writing – review & editing. AA: Writing – review & editing. HR: Writing – review & editing. AI: Writing – review & editing. SL: Writing – review & editing. PS: Writing – review & editing. MS: Writing – review & editing.
